# Association between wrist pain and occupational stress among sewing machine operators

**DOI:** 10.1192/j.eurpsy.2025.1629

**Published:** 2025-08-26

**Authors:** N. Rmadi, A. Hrairi, F. Dhouib, I. Ben Hnia, M. Hajjaji, K. Jmal Hammami

**Affiliations:** 1 Occupational medicine department, Hedi chaker university hospital, University of Sfax, Sfax, Tunisia

## Abstract

**Introduction:**

Given the specific characteristics of their work positions, sewing machine operators (SMOs) represent a vulnerable population to musculoskeletal symptoms such as wrist pain in the leather and footwear sector.

**Objectives:**

This study aims to assess the association between wrist pain and occupational stress among SMOs.

**Methods:**

A cross-sectional study was conducted among SMOs working in a shoe and leather factory. Data collection was carried out using Computer-Assisted Personal Interviews through a pre-established questionnaire. This questionnaire consisted of sociodemographic and professional data. We also assessed occupational stress using the validated French version of Job Content Questionnaire of Karasek.

**Results:**

The average age of SMOs (n = 145) was 35.2 ± 9.9 years, with extremes ranging from 18 to 59 years. A female predominance was noted (sex ratio of 0.25). The average seniority in the current position was 14.4 ± 9.9 years. More than half of the population had high psychological demand, low decision latitude and low social in respectively 53.2%, 97.1% and 67.6%. Among SMOs, 76.3% had wrist pain. In bivariate analysis, wrist pain was positively associated to low decision latitude (p=0.033, OR=2.49, 95%IC [1.11-5.59]. Moreover, it was positively associated to professional seniority (p=0.014).

**Conclusions:**

This study highlights a significant prevalence of wrist pain among SMOs in the leather and footwear sector. It can be exacerbated by various factors, including occupational stress. Addressing these factors is essential for enhancing both the health and productivity of this vulnerable workforce.

**Disclosure of Interest:**

None Declared

